# Combined Effects of Acute Temperature Change and Elevated *p*CO_2_ on the Metabolic Rates and Hypoxia Tolerances of Clearnose Skate (*Rostaraja eglanteria*), Summer Flounder (*Paralichthys dentatus*), and Thorny Skate (*Amblyraja radiata*)

**DOI:** 10.3390/biology8030056

**Published:** 2019-07-26

**Authors:** Gail D. Schwieterman, Daniel P. Crear, Brooke N. Anderson, Danielle R. Lavoie, James A. Sulikowski, Peter G. Bushnell, Richard W. Brill

**Affiliations:** 1 Virginia Institute of Marine Science, College of William and Mary, Gloucester Point, VA 23062, USA; 2 Marine Sciences Department, University of New England, Biddeford, ME 04005, USA; 3 Department of Biology, Marine Biology, and Environmental Science, Roger Williams University, Bristol, RI 02809, USA; 4 School of Mathematical & Natural Sciences, Arizona State University, Glendale, AZ 85306, USA; 5 Department of Biological Sciences, Indiana University South Bend, South Bend, IN 46615, USA

**Keywords:** respirometry, climate change, aerobic scope, multi-stressor, metabolism

## Abstract

Understanding how rising temperatures, ocean acidification, and hypoxia affect the performance of coastal fishes is essential to predicting species-specific responses to climate change. Although a population’s habitat influences physiological performance, little work has explicitly examined the multi-stressor responses of species from habitats differing in natural variability. Here, clearnose skate (*Rostaraja eglanteria*) and summer flounder (*Paralichthys dentatus*) from mid-Atlantic estuaries, and thorny skate (*Amblyraja radiata*) from the Gulf of Maine, were acutely exposed to current and projected temperatures (20, 24, or 28 °C; 22 or 30 °C; and 9, 13, or 15 °C, respectively) and acidification conditions (pH 7.8 or 7.4). We tested metabolic rates and hypoxia tolerance using intermittent-flow respirometry. All three species exhibited increases in standard metabolic rate under an 8 °C temperature increase (Q_10_ of 1.71, 1.07, and 2.56, respectively), although this was most pronounced in the thorny skate. At the lowest test temperature and under the low pH treatment, all three species exhibited significant increases in standard metabolic rate (44–105%; *p* < 0.05) and decreases in hypoxia tolerance (60–84% increases in critical oxygen pressure; *p* < 0.05). This study demonstrates the interactive effects of increasing temperature and changing ocean carbonate chemistry are species-specific, the implications of which should be considered within the context of habitat.

## 1. Introduction

Marine climate change is multidimensional and includes (but is not limited to) rising temperatures, increasing severity and frequency of hypoxic events, and ocean acidification (OA) [1,2,3,4]. These three environmental changes result in interactive, yet poorly understood impacts on both individuals and populations [5,6]. Increases in temperature alone are associated with reduced aerobic scope, and thereby reduced fitness [7,8]. Hypoxia likewise reduces fitness and can cause mortality events [9], while OA can impact various aspects of a species’ biology from behavior to growth rates [10]. A comprehensive understanding of the impacts of climate change on species and populations is, therefore, required both to manage fisheries effectively and to conserve ecologically and economically important resources [11,12,13].

Because it is impossible to fully incorporate the complexity of ecological interactions (e.g., interspecific interactions, regional population distributions, seasonal variability) in models designed to predict the effects of climate change, researchers have attempted to assess vulnerability or resilience using other methods. Testing environmental tolerances, and thus potential for resilience in the face of climate change, is commonly done using aerobic metabolic rate as a proxy for fitness [2,7,14]. Intermittent-flow respirometry measures rates of oxygen consumption that can be used to calculate a range of metabolic parameters including standard metabolic rate (SMR), maximum metabolic rate (MMR), absolute aerobic scope (AS_a_), factorial aerobic scope (AS_f_), and critical oxygen level [15]. Critical oxygen level (S_crit_) is the lowest oxygen level at which SMR remains stable, and below which metabolic rate declines in step with decreases in ambient oxygen [16]. The critical oxygen level can be measured in terms of percent saturation, oxygen content, or partial pressure and is denoted S_crit_, C_crit_, or P_crit_ respectively depending on the units used [17]. Combined, metabolic parameters can assess species- or population-specific tolerances to environmental perturbations, such as those associated with climate change [11,18,19,20]. According to the theory of oxygen- and capacity-limited thermal tolerance (OCLTT) [21], for example, aerobic scope will decline at sub-optimal temperatures because the ability of the cardio-respiratory system to supply oxygen to tissues is reduced. The applicability of this theory is still debated [22], measuring aerobic scope likely can provide information on species-specific physiological abilities and tolerances necessary to predict the effects of shifting environmental conditions [23,24].

Most research concerning the effects of environmental stressors on marine organisms has focused on temperature or pH changes projected to occur over the next 50–100 years in the open ocean [25]. Such studies must, however, reconcile the uncertainty in environmental forecasting with the difficulty of accounting for transgenerational effects [26], the ability of species to alter their distributions [27], and localized adaptation [5]. Additionally, while many perturbation experiments have focused on the specifics of various laboratory treatments, less attention has been given to the natural short-term fluctuations in environmental conditions encountered by coastal species throughout their range or over ontogeny [12,28,29].

Estuarine environments exhibit regular, acute hypoxia and pH variability [30,31], and species inhabiting these environments likely possess the physiological abilities necessary to withstand a broad range of environmental conditions [32], potentially providing some degree of resiliency to the environmental shifts associated with climate change. While there is evidence that species living in variable habitats (such as rocky intertidal pools) are already living near the limits of their physiological capabilities [33], little research has been done to explicitly compare the tolerances of fishes from variable estuarine environments to those from more stable habitats (e.g., higher latitudes or deeper waters) [28]. Species (or populations) inhabiting variable temperate environments tend to be eurythermal, whereas species (or populations) occupying high latitudes tend to be stenothermal [34,35], likely because of the relatively narrow range of temperatures encountered by any given individual [36]. As temperature has a large impact on the metabolism of ectotherms [37,38,39], a comparison between the thermal tolerances of species inhabiting variable and stable habitats may provide insight into species-specific physiological abilities [6,28].

The east coast of the United States includes habitats that differ greatly with respect to their environmental variability [29,31,40,41]. In the mid-Atlantic, inshore species often utilize salt marsh lagoons during the summer, where daily oscillations of ±5 °C are frequently accompanied by fluctuations in pH (±0.5 pH units) and dissolved oxygen (±4.5 mg L^−1^) [31,42]. As climate change effects continue to manifest, these environments are likely to experience even greater swings in temperature, pH, and dissolved oxygen (DO) [43,44,45], which are likely to affect fish species such as the clearnose skate (*Rostaraja eglanteria*) and summer flounder (*Paralichthys dentatus*). Clearnose skate range from the Gulf of Mexico to Cape Cod, USA [46], and are common in the tidal lagoons along the mid-Atlantic. They occur over a temperature range from ~9–30 °C, but prefer ~9–21 °C [47,48]. Summer flounder utilize near-shore regions during the summer, but migrate offshore to spawn in the fall and prefer temperatures between 9 and 24 °C [49,50]. In contrast, the Gulf of Maine is a more stable environment [40], with benthic temperature fluctuations limited to ~3 °C over the entire year [51]. Thorny skate (*Amblyraja radiata*) inhabit the Gulf of Maine and are most abundant between 1 and 5 °C [52], thus occupying a habitat that is very different from that (at least during the summer months) of clearnose skate or summer flounder.

We thus sought to quantify the effects of acute temperature change and elevated *p*CO_2_ levels on the aerobic metabolic rates and hypoxia tolerances of clearnose skate, summer flounder, and thorny skate. This approach allowed us to compare the physiological abilities of sympatric elasmobranch and teleost species (clearnose skate and summer flounder, respectively), as well as allopatric elasmobranch species (clearnose and thorny skates).

## 2. Materials and Methods

All capture, handling, and experimental protocols were approved by the College of William and Mary and University of New England Institutional Animal Care and Use Committee (IACUC-2017-03-14-11935-rwbril and IACUC-012418-003, respectively). Clearnose skate and summer flounder were collected from the Eastern Shore of Virginia using rod and reel during the summers of 2016 and 2017, and maintained in recirculating systems at the Virginia Institute of Marine Science (VIMS) Eastern Shore Laboratory at 20–22 °C. Thorny skate were collected from the Gulf of Maine using a commercial otter trawl [53] in February 2018, and maintained in flow through systems at the seawater laboratory at the University of New England at 5, 9, or 13 °C. All individuals were given at least two weeks to acclimate to captivity before use in experimental trials and were fed *ad libitum* every 2–3 days. Individuals were fasted for 48 h prior to use in an experiment to ensure they were in a post-absorptive state [54].

A total of 24 clearnose skates and 17 thorny skates were subjected to three to four trials each, resulting in eight trials at each of the three temperatures representing the mid- to upper-range of thermal tolerances (20, 24, 28 °C for clearnose skate; 5, 9, 13 °C for thorny skate), under two CO_2_ conditions representing the present day and that predicted to occur by the end of the century (pH of 7.8 and 7.4, respectively) [55,56]. A total of eight summer flounder were subjected to two trials each at 22 and 30 °C under elevated *p*CO_2_ conditions (pH 7.4). Because the most extreme treatment (28 °C and elevated *p*CO_2_) resulted in 40% mortality in preliminary trials on clearnose skate, this treatment was discontinued. The equivalent treatment (13 °C and elevated *p*CO_2_) of thorny skate experiments was likewise excluded.

Clearnose and thorny skates were acclimated to trial conditions for 48 h and one week, respectively. For clearnose skate, MMR was obtained using an established chase protocol involving enforced exercise (i.e., chasing and turning individuals over to induce swimming until they no longer responded to tactile stimulus) followed by one minute of air exposure [5]. Thorny skate respond to being handled by curling into a ball, and thus could not be chased. Instead, this species was air exposed for eight minutes. Respirometry protocols used with summer flounder were modified from those of Capossela et al. [54], in that fish were not fitted with additional sensors to measure exhalent oxygen. As Capossela et al. [54] only measured SMR and P_crit_, those were the only variables measured from the summer flounder here. These were calculated as described below. We thus re-analyzed the data from Capossela et al. [54] along with the elevated *p*CO_2_ data we collected for this study.

Following the chase and/or air exposure protocols, individuals were placed in custom-built Plexiglas respirometers constructed to ensure that the volume to animal mass ratio fell between 30:1 and 50:1. The chambers were equipped with fiber optic oxygen sensors and a recirculating pump, as recommended by Rogers et al. [57]. The respirometry chambers were placed in an outer water bath from which water was used to flush the respirometer. A computer program (developed in Dasylab 13.1; National Instruments, www.ni.com) logged data and continuously controlled temperature and oxygen levels for 36 h. Each measurement cycle lasted 10 min and began with a 5 min flush cycle, which was terminated when the flush pump was turned off. After allowing 2 min for the water in the measurement system to mix, the decline in oxygen over a 5 min period was recorded before the flush pump was turned on again. At no time during normoxic trials was the chamber oxygen level allowed to fall below 80%. At the end of each data recording interval, the Dasylab software executed a call to an Excel macro routine that calculated the rate of change of O_2_ content (converted from percent saturation) with time (Δ[O_2_] × t^−1^) based on a linear regression of the recorded oxygen levels against elapsed time (t). The Excel macro routine subsequently calculated MO_2_ as follows:

MO_2_ = (Δ [O_2_] × t^−1^) × V × W^−1^ (1)

where *V*—respirometer volume (l) corrected for fish volume and *W*—fish mass (kg). To estimate microbial oxygen consumption, rates of O_2_ depletion were measured both before and after the trial (i.e., when the fish was not in the chamber). We used linear regression to estimate rates of oxygen depletion due to microbial respiration occurring over the time course of an experiment. These values were then subtracted from the measured rates of oxygen decline.

**Figure 1:**
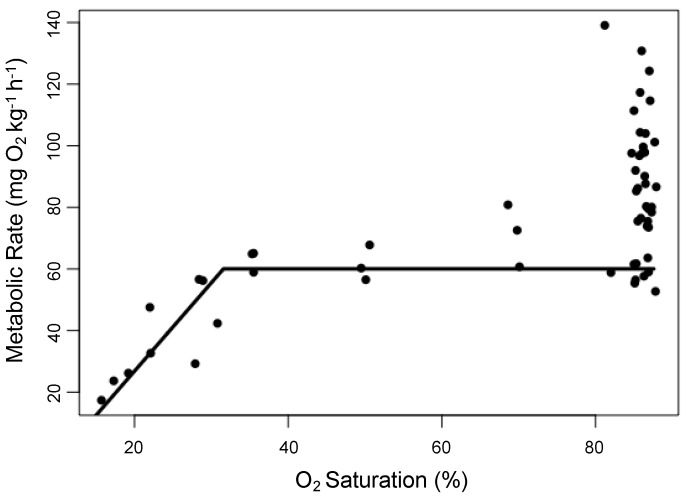
Data from a single respirometry trial. S_crit_ is the intersection of the two linear regression lines.

MMR was taken as the single highest metabolic rate measured during the first 12 h following the chase and/or air exposure protocols. For all three species, SMR was taken as the mean of the lowest 10 metabolic rates during the middle 12 h of the trial. Aerobic scope was calculated in two ways (AS_a_ = MMR − SMR; AS_f_ = MMR × SMR^−1^). Following SMR measurements, oxygen was reduced in a step-wise fashion, with three measurements taken at 80, 60, 40, 30, 20, and 10% O_2_ saturation. Trials were terminated when MO_2_ dropped to zero and individuals were then allowed to recover in fully oxygenated seawater for 1 h before being returned to holding tanks. S_crit_ was defined as the point at which an individual could no longer maintain SMR. This was done following Schurmann and Steffensen [58], where metabolic rate measurements below SMR were evaluated to determine if all subsequent values were also below SMR. When this was true, this subset of points was fit with a linear regression. The oxygen saturation where this regression line and SMR intersected was defined as S_crit_ (Figure 1). From these, we calculated the critical oxygen content (C_crit_) by converting the percent saturation to mg L^−1^ using known temperature and salinity values. We also calculated critical oxygen partial pressure (P_crit_) by determining the partial pressure of oxygen at 1 atmosphere on the day of the start of the trial, calculating the percent saturation of seawater, and multiplying that by the temperature- and salinity-specific oxygen content of seawater at full saturation.

To better compare across different temperature ranges, we calculated Q_10_ values for SMR as follows:

(2) $$Q_{10}=(R_{2}÷R_{1}){}^{10}÷(T_{1}-T_{2})$$

where *Q*_10_ is the temperature coefficient for SMR, *R*_1_ is the SMR at *T*_1_, and *R*_2_ is the SMR at *T*_2_.

To increase pCO_2_, and thus reduce pH, we used the standard method of bubbling CO_2_ gas [59]. A stand-alone system (TUNZE 7074; Penzberg, Germany), connected to a laboratory-grade glass pH probe in the outer water bath, controlled an electronic solenoid valve connected to a cylinder of CO_2_. The system injected a slow stream of CO_2_ into the outer water bath whenever pH of the seawater rose above the set point. Using this method, it was possible to maintain pH within ±0.05 units of the desired level, and it was unlikely that there was a biologically significant gradient in seawater pH within the tanks, as the outer water bath was continuously mixed by a submersible pump.

The pH of each outer water bath in the low pH treatment was independently validated at the start and end of each trial using a SDL100 pH meter (Extech Instruments, Nashua, NH, USA) calibrated daily with fresh pH buffers (Tunze, Penzberg, Germany). Additionally, water samples were taken at the start and end of each trial for dissolved inorganic carbon (DIC) and total alkalinity (TA) Automated InfraRed Inorganic Carbon Analyzer (AIRICA) and Metrohm analyses, or for spectrophotometric determination of pH and TA Metrohm analysis [60]. All pH values were subsequently calculated from these additional measurements using the CO2SYS software [61] with the constants K1 from Mehrbach et al. [62] (refit by Dickson and Millero [63]), and KHSO_4_ from Dickson et al. [60]. Data on the seawater chemistry of present day *p*CO_2_ trials were obtained from 10 samples taken from the water inflow to the seawater labs in Virginia and Maine (Table 1). Equivalent seawater chemistry data for the summer flounder experiments performed by Capossela et al. [54] were not available.

All statistical analyses were conducted using SAS 9.4 (SAS Institute, Cary, NC, USA). Data were analyzed using a multivariate repeated measures analysis of variance (ANOVA) using the MIXED procedure to account for the correlation between metabolic indices, with individual being the random factor upon which multiple measures were made [64]. SMR, MMR, AS_a_, and P_crit_ were considered response variables, and temperature, *p*CO_2_ level, and a dummy variable representing the number of repetitions being measured on a single individual were considered factors. We modeled the heterogeneity in responses among temperature treatments and specified the Kenward–Roger method for calculating the degrees of freedom [65]. Model selection between different variance/covariance structures was performed using Bayesian information criterion (BIC,) and significant differences were determined using 95% confidence intervals derived using the least squares means (LSM) estimate statement in SAS. Model data are presented from model structures using compound symmetry correlation structures. All statistics were evaluated with a significance level of α = 0.05.

## 3. Results

Our water chemistry values (Table 1) were largely consistent with published values for both the Chesapeake Bay [66,67] and the Gulf of Maine [68,69]. The elevated *p*CO_2_ treatment had higher calculated *p*CO_2_ values than expected [66,69].

We collected data from 24 clearnose skate (1.3 ± 0.06 kg; mean mass ± standard error, 17 thorny skate (1.4 ± 0.2 kg), and 9 summer flounder (0.36 ± 0.01 kg), and re-analyzed data from 9 summer flounder reported by Capossela, et al. [53]. Despite high variability within any given parameter, our models revealed differences among the metabolic response to changing environmental parameters. Model-derived estimates for all parameters can be found in Table S1.

### 3.1. SMR and Q_10_

**Figure 2:**
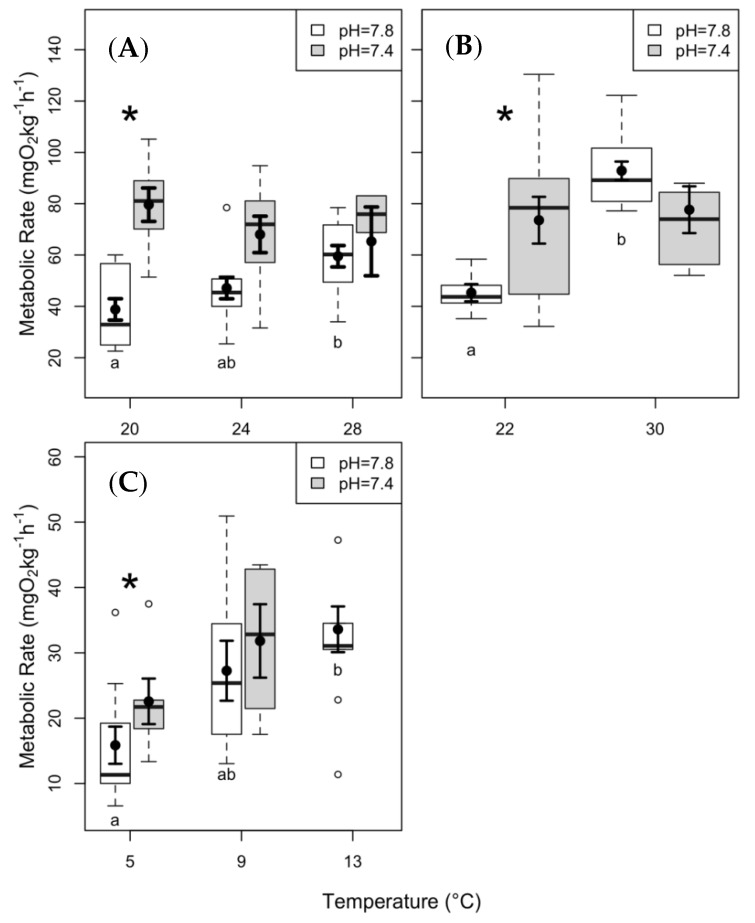
Standard metabolic rate (SMR) data from (**A**) clearnose skate, (**B**) summer flounder, and (**C**) thorny skate. Box and whisker plots represent raw data, with whiskers representing maximum and minimum points within 1.5 times the interquartile range above the upper quartile and below the lower quartile. Open circles denote points outside of this range, while the filled circles and lines indicate the model-derived estimates and standard errors for each treatment condition. The asterisks above the boxplots represent significant differences between pH treatments within a given temperature. The letters below the boxes represent significant differences among temperatures within a given pH level.

We observed an increase in SMR with increasing temperature in both skate species during the present day *p*CO_2_ (i.e., at high pH) experiments (Figure 2A,C; Table 1). In clearnose skate, SMR was significantly higher under the elevated *p*CO_2_ at 20 °C and 24 °C (Figure 2A). SMR of summer flounder significantly increased between 22 °C and 30 °C (Figure 2B; *p* < 0.01), and elevated *p*CO_2_ caused SMR to increase at 22 °C (*p* < 0.01). There was no significant effect of temperature on SMR under elevated *p*CO_2_ (Figure 2B). In contrast, thorny skate did not demonstrate any significant differences in SMR elevated *p*CO_2_ at either test temperature (Figure 2C).

To facilitate comparisons across species, we compiled Q_10_ values for SMR for all three species (Table 2). Clearnose skate and summer flounder had relatively low Q_10_ values at the present day *p*CO_2_ treatment, while thorny skate Q_10_ was more similar to the expected value between 2 and 3 [8,14]. Under elevated *p*CO_2_, we observed that clearnose skate and summer flounder Q_10_ values more than doubled, while thorny skate Q_10_ increased to a lesser degree.

### 3.2. Maximum Metabolic Rate and Aerobic Scope

**Figure 3:**
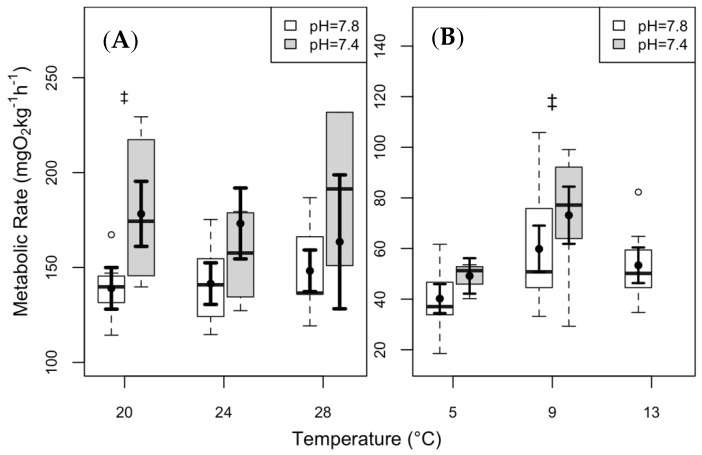
Maximum metabolic rate (MMR) of (**A**) clearnose skate and (**B**) thorny skate. Box and whisker plots represent raw data, with whiskers representing maximum and minimum points within 1.5 times the interquartile range above the upper quartile and below the lower quartile. Open circles denote points outside of this range, while the filled circles and lines indicate the model-derived estimates and standard errors for each treatment condition. There were no significant differences in any of the pairwise comparisons, but the “‡” symbol denotes near significance (*p* = 0.051 in clearnose skate, and *p* = 0.07 in thorny skate).

The differences in mean MMR at 20 °C in clearnose skate were nearly significant between the two *p*CO_2_ conditions (Figure 3A; *p* = 0.051). Thorny skate showed an increasing trend in MMR within a given temperature (at 9 °C) at the elevated *p*CO_2_ (Figure 3B; *p* = 0.07).

**Figure 4:**
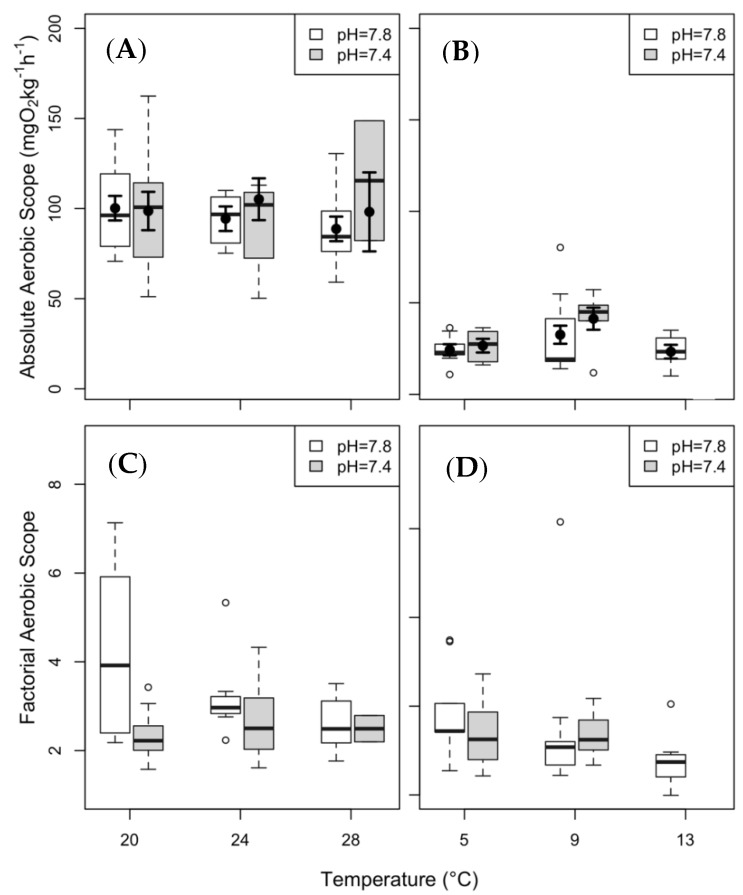
Aerobic scope (AS) of clearnose skate (panels (**A**,**C**) and thorny skate (panels (**B**,**D**). AS_a_ is presented in panels (**A**,**B**), and AS_f_ in panels (**C**,**D**)). Box and whisker plots represent raw data, with whiskers representing maximum and minimum points within 1.5 times the interquartile range above the upper quartile and below the lower quartile. Open circles denote points outside of this range, while the filled circles and lines indicate the model-derived estimates and standard errors for each treatment condition. AS_a_ was analyzed for model analysis, but AS_f_ was not.

The AS_a_ of clearnose skate did not vary significantly under any of the treatment conditions. The AS_a_ of thorny skate was significantly higher at 5 °C under elevated *p*CO_2_. To compare results between species, and following the recommendations of Clark et al. [7] and Lapointe et al. [64], we have included plots for AS_a_ and AS_f_ in Figure 4. Trends between the two different metrics are similar, although no statistical analysis was performed on AS_f_, as the two metrics were too similar to be fitted by the model.

### 3.3. Hypoxia Tolerance

**Figure 5:**
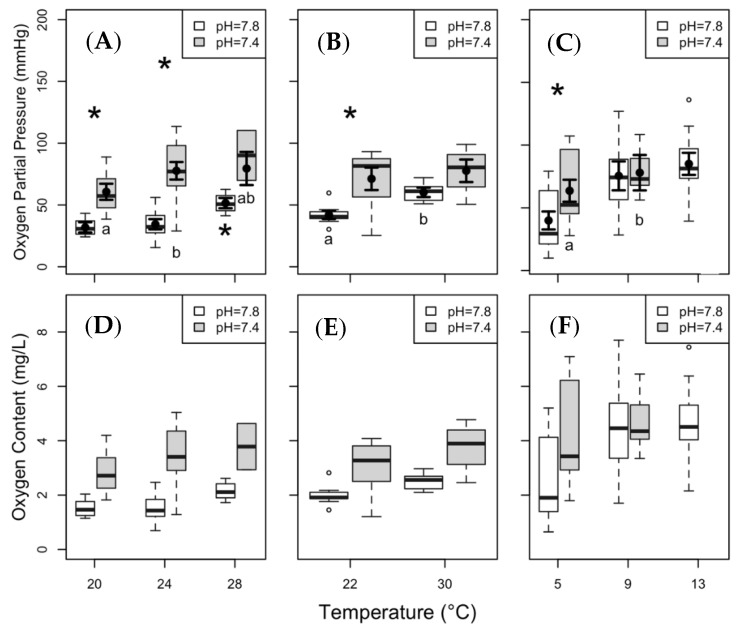
Critical oxygen partial pressure (P_crit_) and critical oxygen content (C_crit_) of clearnose skate (panels (**A**,**D**)), summer flounder (panels (**B**,**E**)), and thorny skate (panels (**C**,**F**)). Box and whisker plots represent raw data with whiskers representing maximum and minimum points within 1.5 times the interquartile range above the upper quartile and below the lower quartile. Open circles denote points outside of this range, while the filled circles and lines indicate the model-derived estimates and standard errors for each treatment condition. Asterisks above the boxes indicate significant differences between the pH treatments within a given temperature, while asterisks and letters below the boxes indicate significance across temperatures within a pH treatment. No statistical analyses were run on C_crit_ and these graphs are included only as an aid for comparison to other studies.

The hypoxia tolerance of clearnose skate under present day *p*CO_2_ (i.e., high pH) was reduced at increased temperature, as shown by a significantly higher P_crit_ at 28 °C compared with 20 °C and 24 °C (*p* < 0.01 for both). Under elevated *p*CO_2_, we observed a significant increase in P_crit_ between 20 °C and 24 °C (*p* = 0.04). Clearnose skate exhibited marked reductions in hypoxia tolerance under elevated *p*CO_2_ (Figure 5A), with significant elevations in P_crit_ at 20 °C and 24 °C (*p* < 0.01 for both). Summer flounder showed the expected significant increase in P_crit_ under elevated temperatures at present day *p*CO_2_ (*p* < 0.01), as well as a significant increase under elevated *p*CO_2_ at 22 °C (*p* < 0.01; Figure 5B). The P_crit_ of thorny skate at 13 °C was significantly higher compared with that measured at 5 °C under present day *p*CO_2_ (*p* = 0.04); and P_crit_ at 5 °C was significantly higher at elevated *p*CO_2_ than under present day *p*CO_2_ (*p* < 0.01; Figure 5C).

## 4. Discussion

Our study compares the environmental tolerances of species in two disparate environments under projected conditions (elevated temperature and elevated *p*CO_2_), focusing on how observed tolerances are impacted by multiple, concurrent stressors. In general, the physiological abilities to withstand acute exposure to environmental stress were more similar between the sympatric species (clearnose skate and summer flounder) than the abilities of the allopatric species (thorny skate).

Although the *p*CO_2_ values used for the elevated pCO_2_ treatment were somewhat variable, targeted pH values were maintained. For the purposes of interspecific comparison, moreover, the difference between the present day and elevated *p*CO_2_ treatments is likely more important than the actual values. The variability observed in the present-day conditions is likely a result of the natural conditions in near-shore water pumped in the seawater facilities; additional manipulation of the carbonate chemistry of the seawater was deemed cost-prohibitive, and likely unwarranted as the modified seawater would not mimic estuarine conditions.

The SMR values measured at present-day *p*CO_2_ levels (38.8 ± 4.2 mg O_2_ kg^−1^ h^−1^ at 20 °C for clearnose skate; 45.3 ± 3.4 mg O_2_ kg^−1^ h^−1^ at 22 °C for summer flounder; 15.9 ± 2.8 mg O_2_ kg^−1^ h^−1^ at 5 °C for thorny skate; Figure 2) were lower than other studies at similar temperatures (e.g., 100–150 mg O_2_ kg^−1^ h^−1^ at 20 °C [70]; 68–84 mg O_2_ kg^−1^ h^−1^ at 10 °C [71]), although this could be attributed to the demersal nature of these study species. The increase in SMR under elevated *p*CO_2_ within the lowest test temperatures for all three species (105%, 64%, and 43% increase for clearnose skate, summer flounder, and thorny skate, respectively), and the declining difference in SMR between *p*CO_2_ treatments at elevated temperatures (down to 10%, −16%, and 17% for clearnose skate, summer flounder, and thorny skate, respectively; Figure 2) matches trends from little skate (*Leucoraja erinacea*) exposed to elevated temperatures and *p*CO_2_ [5], but differs from similar studies on other species [72,73,74,75]. This suggests there may be conserved physiological mechanisms driving this response, but much is still unknown regarding the mechanisms underpinning the observed patterns. The results presented here may be due to bradycardia, increased ventilatory rates, and increased blood pressure [76,77], or to the increased metabolic cost of buffering against plasma pH changes [14,78] and increased ion transport [24]. These known physiological stresses are unlikely, however, to increase the metabolic rate to the extent we observed in this study. An alternative explanation is that individuals are more active under elevated *p*CO_2_ (i.e., low pH) conditions [79].

The effect of elevated *p*CO_2_ on SMR masks the temperature effect observed under present-day conditions, indicating that the responses to these stressors are not additive. This may be the result of an alternative version of the OCLTT hypothesis, where the physiological consequences of elevated *p*CO_2_ (rather than temperature) are predicted to limit oxygen delivery [21,80]. Different responses to temperature under present-day conditions compared to under elevated *p*CO_2_ suggest that there are interactive mechanisms regulating oxygen delivery in fishes [8,10,24,81]. Elevated plasma levels of CO_2_ (with concomitant reductions in plasma pH) reduce hemoglobin oxygen affinity (Bohr effect) and maximum blood oxygen content (Root effect); although the extent of these is unknown in the study species. Alternatively, the effects of one stressor could be compensating the effects of the other [24], resulting in the masking effects. For example, increased metabolic costs of acid-base regulation under ocean acidification could be offset by reduced energetic demand elsewhere. This phenomenon has been demonstrated with low pH-induced metabolic depression in isolated gill cells [82]. Given the large knowledge gaps concerning the mechanisms underpinning our results, we argue—as have others—that more multi-stressor studies are needed [12,13,83,84,85].

Clearnose skate and summer flounder exhibited lower Q_10_ values (Q_10_ = 1.62 and 1.07, respectively) at present day *p*CO_2_ than the thorny skate (Q_10_ = 3.87). While Q_10_ values lower than 2 have been associated with a decreasing ability to function [86], for the two Mid-Atlantic estuarine species studied here (i.e., clearnose skate and summer flounder), the low Q_10_ values are rather indicative of the ability to maintain a consistent level of aerobic ATP production over a relatively broad range of temperatures [37,87], potentially signifying resilience to the coastal warming predicted under climate change [23]. High Q_10_ values, in contrast, have been attributed to species from stable environments [88]. The thorny skate, therefore, may not possess isozymes (or the genetic plasticity to produce isozymes) that reduce the effects of temperature on metabolic rate over a broad range of temperatures [32,86,89], and may thus be more sensitive to temperature increases than the other two study species. The idea that the effects of temperature on metabolic rate are closely associated with native thermal range [37], has received mixed support from other studies looking specifically at different populations or species. For example, Di Santo [5] found increased sensitivity to temperature in more northern populations of little skate. According to the evolutionary trade-off hypothesis [90], the resting metabolic rate of a species (or population) at over its normal environmental temperature range represents an evolutionary optimization. In other words, species- or population-specific optimization of metabolic rates to a given temperature (or range of temperatures) might not be explained purely through the kinetic energy of sub-cellular constituents, but may rather be a suite of complex tradeoffs [86,90,91]. This becomes evident as all three species exhibited a decrease in Q_10_ under elevated *p*CO_2,_ driven by increases in SMR and emphasizing the masking impact of this additional stressor. Under projected climate change scenarios, elevated temperatures and ocean acidification are likely to have interactive effects on cellular processes [37,39,92,93]. While the Q_10_ values presented here offer some insight into species-specific sensitivity, more research on the interactive effects of multiple, concurrent stressors on metabolism is needed.

We did not observe any significant trends in MMR between the two skate species with either temperature or *p*CO_2_ (Figure 3). This may be attributable to an insufficient stressor prior to the respirometry trial. Although the values presented here are lower than published values for other fish species measured at similar temperatures [20,70,94], this could also be attributed to the more sedentary life style of our study species. Alternatively, the Fry paradigm [38] for diminishing MMR values above an optimal temperature may not hold in these species [14]. There is widespread dissent in the literature regarding the appropriate methods to obtain MMR [14,95] and whether the standard Fry paradigm is valid, which make us hesitant to draw more definitive conclusions.

The aerobic scope data do not support the existence of a bell-shaped curve centered on a single optimal temperature (T_opt_), but rather AS being relatively temperature-independent. These results may be driven by multiple T_opt_ values for different physiological processes [7,36], and are consistent with other studies [14,26,74,96,97,98]. The lack of significant reduction in aerobic scope under high stress conditions suggests that clearnose and thorny skates, may exhibit resilience to climate change in their respective environments. Given that there have been conflicting reports on the capacity of elasmobranch species to acclimate to climate change conditions [99,100], the findings of this study represent an important step in understanding the physiological tolerances of this understudied group [101]. An important caveat is that because we used wild-caught adults, any early life history detriments to condition and survival [100,102,103] remain unmeasured.

Our most significant finding, however, may be that clearnose skate (P_crit_ 32 ± 2 mmHg at 20 °C; mean ± SE) are as hypoxia tolerant as epaulette shark (P_crit_ 38 mmHg at 28 °C); and the latter have been deemed to have exceptional hypoxia tolerance [104,105]. While the physiological mechanisms underlying the hypoxia tolerance of epaulette shark have received considerable attention [73,106,107,108,109,110], there are no equivalent data for clearnose skate, and we encourage studies in this area. Our P_crit_ data show, however, that summer flounder are also tolerant to hypoxia (P_crit_ = 42 mmHg at 22 °C). Considering the correlations between hypoxia tolerance and the environmental variability of a species’ native habitat, we note that epaulette sharks live in reef and tidal environments that experience large diel and tidal cycle fluctuations in temperature, oxygen, and pH, similar to the changes occurring in estuaries along the U.S. mid-Atlantic [29,30,111]. Other species from variable environments are also hypoxia tolerant, including blue crabs (*Calinectus sapidus*) [112] and crucian carp (*Carassius carassius*), as well as many rocky tidepool fishes [33]. These results, however, are not ubiquitous. Sandbar shark (*Carcharhinus plumbeus*) have a markedly higher P_crit_ value than clearnose skate or summer flounder [17], despite being a sympatric species. As sandbar shark are an obligate ram-ventilating species [39], this difference is unsurprising and is supported by findings on bonnethead shark (*Sphyrna tiburo*), which live in seagrass meadows likely to experience large diel cycles in dissolved oxygen. In contrast to clearnose skate and summer flounder, thorny skate are relatively intolerant to hypoxia (P_crit_ = 75 mmHg at 9 °C), most likely because this species occupies the Gulf of Maine, an environment that does not exhibit wide swings in temperature and oxygen levels [40,51]. Similarly, the shovelnose ray (*Aptychotrema rostrata*) that occupies an environment where it rarely encounters hypoxia [113] has a P_crit_ = 54 mmHg at 28 °C [104].

The increases in P_crit_ under elevated *p*CO_2_ (84%, 69%, and 60% increases in P_crit_ for clearnose skate, summer flounder, and thorny skate, respectively) may be the result of the inability of the non-bicarbonate blood buffering capacity of all three study species to limit reductions in plasma pH (and subsequently the intracellular environment) under elevated *p*CO_2_. To the best of our knowledge, there is no information regarding intracellular pH (pH_i_) of elasmobranch red blood cells following exposure to simulated OA. Studies on brain, white muscle, and liver tissue isolated from teleost fishes and exposed to elevated *p*CO_2_ have, however, found either no change or an increases in pH_i_ [24,72,81,114], suggesting OA may not have a negative impact blood oxygen transport. This is supported by a lack of increase in hematocrit following exposure to elevated *p*CO_2_ [74,114,115]. As the differences in P_crit_ were most apparent in the mid-Atlantic species, further work on in vivo blood pH levels under changing *p*CO_2_ conditions, as well as quantification of the changes in blood oxygen affinity (Bohr shift) and maximum oxygen carrying capacity (Root effect), in these species would help elucidate the mechanisms underpinning our observed reductions in hypoxia tolerance. Because of the high hypoxia tolerance of clearnose skate, we predict that this species may also demonstrate a high blood oxygen affinity and a large Bohr effect, similar to that seen in the hypoxia tolerant bat ray (*Myliobatis californica*) [104,116]. We also expect summer founder blood to have similar physiological characteristics to those of blood from European flounder (*Platichthys flesus*) [117].

These mechanisms are largely speculative, however, as there are conflicting reports of the effects of elevated *p*CO_2_ on hypoxia tolerance. For example, epaulette sharks do not exhibit decreases in hypoxia tolerance under elevated *p*CO_2_ conditions [73]. This may be attributed to the chronic (60-day) exposure of epaulette shark to elevated *p*CO_2_ conditions, compared with the acute exposures we employed. Other studies have reported increases in P_crit_ under elevated *p*CO_2_ in European eel (*Anguilla Anguilla)* [118] and European flounder [119]; and in acidified water for rainbow trout (*Salmo gairdneri*) and carp (*Cyprinus carpio*) [120]. These results are, however, not universal [57], as croaker (*Leiostomus xanthurus)* and mummichog (*Fundulus heteroclitus)* exhibit no change in P_crit_ under elevated pCO_2_ [43,57]. We note, however, these two species are common occupants of mid-Atlantic estuaries, and thus regularly experience elevated *p*CO_2_ conditions [43].

From an ecological perspective, the observed effect of elevated *p*CO_2_ on hypoxia tolerance is concerning. Currently, clearnose skate and summer flounder are unlikely to encounter waters below their P_crit_, assuming the water is at a pH of 7.8 [12,28,30]. Because of the effects of climate change, however, individuals in coastal waters are more likely to experience concurrent hypoxia and elevated *p*CO_2_ [31,56,121,122]. While estuarine and coastal species may be able to tolerate current conditions, further extremes of these parameters may force populations to move to alternative habitats. While at present, it is unlikely that thorny skate regularly encounter hypoxia, warming shelf waters could induce changes in dissolved oxygen distribution, resulting in unfavorable habitats in areas such as the Gulf of Maine [40,51,123]. Activity patterns observed in dogfish (*Scyliorhinus canicula*) suggest that sluggish benthic elasmobranch species do not increase activity under hypoxic conditions [124], although the more active bonnethead shark does [125]. The sedentary strategy of non-obligate ram ventilating species could, therefore, limit their ability to exploit novel habitats under unfavorable environmental conditions.

Recently, Wood [126] argued that P_crit_ as a metric of hypoxia tolerance is of limited utility owing to numerous factors including the lack of repeatability and consistency and an insufficient theoretical underpinning. He proposed several alternative metrics that could be used in place of P_crit_, including loss of equilibrium or measurements of ventilation. While we agree that alternative measure of hypoxia tolerance can provide useful information, for the purposes of our study of benthic flatfishes, the loss of equilibrium is not a useful metric. Further, because the calculation of P_crit_ was standardized across all three species, concerns regarding different methodology were alleviated. We agree with Regan et al. [127], that “P_crit_ contributes to a more complete picture of an animal’s total hypoxic response by capturing the suite of aerobic contributions to hypoxic survival in a single value”, and hope the data presented here can help further our understanding of hypoxia tolerance in a range of coastal species.

## 5. Conclusions

Understanding the species- and population-specific response to the multiple environmental stressors associated with climate change is essential for managing marine sources in a changing environment. The results presented here quantify the physiological limits of clearnose skate, summer flounder, and thorny skate with respect to acute changes in temperature and elevated *p*CO_2_. All three species exhibited increases in SMR (105%, 42%, and 22% for clearnose skate, summer flounder, and thorny skate, respectively) at the lowest test temperature under elevated *p*CO_2_, and this increases masked increases in SMR at the high test temperatures. All three species also showed decreased hypoxia tolerance (150%, 85%, and 113% increases in P_crit_) under the most extreme combined stressors. While the clearnose skate did exhibit remarkable hypoxia tolerance under the least stressful treatment, as climate change impacts continue to increase in severity, even this species may be pushed towards or past the limits of their physiological capabilities. Incorporating multi-stressor studies into future climate change research is essential to predicting how species will respond to changing environmental conditions. If conditions are near the limits of physiological abilities, individuals may choose to seek out more favorable habitats, resulting in shifting distributions, fecundities, and food web dynamics with cascading ecological and economic implications.

## Figures and Tables

**Table 1:** The carbonate chemistry parameters during the high and low pH (i.e., present day and elevated *p*CO_2_) experiments. Values are averaged across all temperature treatments. All values represent mean ± SD.

Species	*p*CO_2_ Treatment	Salinity (ppm)	pH	Alkalinity (μmol kg^−1^)	*p*CO_2_ (μatm)
Clearnose skate	Present day	30 ± 0.3	7.84 ± 0.02 *	2317 ± 17	703 ± 33 *
	Elevated	30 ± 0.6	7.44 ± 0.04 *	2285 ± 14	2290 ± 262 *
Summer flounder	Present day	31 ± 0.5	Unknown	Unknown	Unknown
	Elevated	29 ± 0.4	7.46 ± 0.06 *	2258 ± 11	2204 ± 301 *
Thorny skate	Present day	33 ± 0.7	7.87 ± 0.04	2151 ± 17	569 ± 57 *
	Elevated	33 ± 0.3	7.45 ± 0.05	2155 ± 10	2111 ± 204 *

* Values were calculated using CO2SYS, rather than being measured directly.

**Table 2:** The effects of temperature on standard metabolic rate measured as Q_10_ values. For both skate species, the values are reported for two different temperature ranges because of the small or non-existent sample size at the highest temperatures and lowered pH level.

		Q_10_	
Species	Temperature	Present Day *p*CO_2_	Elevated *p*CO_2_
Clearnose Skate	20–28 °C	1.71	
	20–24 °C	1.62	0.78
Summer Flounder	22–30 °C	2.45*	1.07
Thorny Skate	5–13 °C	2.56	
	5–9 °C	3.87	2.34

* Values were not explicitly measured or controlled by Capossela et al. [54].

## Supplementary Materials

The following are available online at https://www.mdpi.com/2079-7737/8/3/56/s1, Table S1: The estimated values ± standard error based on model output.
