# Supplementary material for: Combined Effects of Acute Temperature Change and Elevated pCO2 on the Metabolic Rates and Hypoxia Tolerances of Clearnose Skate (Rostaraja eglanteria), Summer Flounder (Paralichthys dentatus), and Thorny Skate (Amblyraja radiata)
Source: Biology (Basel). 2019 Jul 26;8(3):56. doi: 10.3390/biology8030056 (PMC6783964; doi:10.3390/biology8030056)
Supplement: Supplementary file 1 [file biology-08-00056-s001.pdf]

Supplementary Materials:

Combined Effects of Acute Temperature Change and Elevated *p*CO_2 on the Metabolic Rates and Hypoxia Tolerances of Clearnose Skate (*Rostaraja eglanteria*), Summer Flounder (*Paralichthys dentatus*), and Thorny Skate (*Amblyraja radiata*)

Gail D. Schwieterman, Daniel P. Crear, Brooke N. Anderson, Danielle R. Lavoie, James A. Sulikowski, Peter G. Bushnell, and Richard W. Brill

Table 1. The estimated values ± standard error based on model output.

Species	Temp	*p*CO_2	SMR	MMR	ASa	P_{crit}
Clearnose Skate	20 °C	Present	39 ± 4	139 ± 11	100 ± 7	33 ± 4
		Elevated	80 ± 6	178 ± 17	99 ± 11	61 ± 6
	24 °C	Present	47 ± 4	141 ± 11	94 ± 7	34 ± 4
		Elevated	68 ± 7	173 ± 19	105 ± 12	78 ± 7
	28 °C	Present	59 ± 4	148 ± 11	89 ± 7	51 ± 4
		Elevated	65 ± 13	163 ± 35	98 ± 22	79 ± 13
Summer Flounder	22 °C	Present	45 ± 3	-	-	42 ± 3
		Elevated	74 ± 9	-	-	71 ± 9
	30 °C	Present	93 ± 4	-	-	60 ± 4
		Elevated	78 ± 9	-	-	78 ± 9
Thorny Skate	5 °C	Present	16 ± 3	40 ± 6	24 ± 3	40 ± 7
		Elevated	23 ± 3	49 ± 7	27 ± 4	64 ± 9
	9 °C	Present	27 ± 5	60 ± 9	33 ± 5	75 ± 11
		Elevated	32 ± 6	73 ± 11	41 ± 6	78 ± 14
	13 °C	Present	34 ± 3	53 ± 7	23 ± 4	85 ± 9
		Elevated	-	-	-	-
